# The complete mitochondrial DNA sequence of Yimeng scorpion (*Mesobuthus martensii*)

**DOI:** 10.1080/23802359.2019.1703584

**Published:** 2019-12-18

**Authors:** Yan-Zhen Zhang, Yan-Qing Ji, Da-Cheng Kang, Ling-Xiao Liu, Yun-Guo Liu

**Affiliations:** a College of Life Sciences, Linyi University, Linyi, China;; b College of Life Sciences and Technology, Xinjiang University, Urumqi, China;; c Linyi Academy of Agricultural Sciences, Linyi, China

**Keywords:** Yimeng scorpion, *Mesobuthus martensii*, mitochondrial genome

## Abstract

Yimeng scorpion is a specific geographical indication breed of Yimeng Mountain area in China. The complete mitochondrial genome sequence of Yimeng scorpion was determined for the first time (Accession number MN597087). It is mitochondrial genome (14,840 bp) contains 13 protein-coding genes, 21tRNA genes, 2 ribosomal RNA genes and one large non-coding region (a possible control region). Moreover, tRNA-ASP-loss was observed from the Yimeng scorpion mitochondrial genome. The mitochondrial genome sequence of the Yimeng scorpion enriches data resource for further research on genetic mechanism and classification.

There are about 29 species and subspecies of scorpions have been reported in China, among which the most widely distributed is the *Buthus martensii*, also known as *Mesobuthus martensii* (Zhu et al. 2004; Shi and Zhang 2005). Yimeng scorpion is a kind of special economic animal with remarkable medicinal properties and high edible value, mainly dominated by natural reproduction and a few for artificial breeding, which is different from scorpions in other parts of China (Di 2013). Here, the complete mitochondrial genome of Yimeng scorpion was sequenced and characterized in detail. Scorpion sample was collected from Mengyin City (35°43′N, 117°57′E), Shandong Province, China, in June, 2019. The specimen of Yimeng scorpion, named as YimengSC-01 was stored in College of Life Sciences, Linyi University, Linyi, China. Total genomic DNA was extracted from Yimeng scorpion muscle according to (Liu et al. 2012, 2016). Then, the complete mitochondrial genome was sequenced using a shotgun approach and assembly. Whereafter, DNA sequence was analyzed using MEGA 7 (Kumar et al. 2016) and protein-coding genes were analyzed by ORF Finder (http://www.ncbi.nlm.nih.gov/gorf/gorf.html) using the invertebrate mitochondrial code. The tRNA genes were identified by ARWEN (Laslett and Canbäck 2008) and tRNA-scan SE (Lowe and Eddy 1997).

The mitochondrial genome of Yimeng scorpion (Accession number MN597087) was 14,840 in length, of which 14,023 nucleotides are coding DNA, and 817 nucleotides are non-coding DNA. It contains the 13 protein-coding genes, two rRNA genes, 21 tRNA genes and a possible control region. The arrangement and composition of the mitochondrial genome is also comparable to the case of other Arthropoda (Lavrov et al. 2000; Masta and Boore 2004; Hwa Choi et al. 2007). A phylogenetic tree was constructed based on the comparison of Yimeng scorpion mitochondrial genome sequences with other Buthidae species using neighbor-joining method (Figure 1). There are found 23 overlapping regions (total 168 bp) and 7 intergenic spacers (total 38 bp) among the genes. The total base composition of the mitochondrial genome is 29.58% A, 36.88% T, 12.15% C and 21.38% G, and an A + T (66.46%)-rich feature occurs in the Yimeng scorpion. To investigate the nucleotide bias, skew for a given strand was calculated as (A-T)/(A + T) or (G-C)/(G + C) (Perna and Kocher 1995). The AT and GC skews for the Yimeng scorpion mitochondrial genome were −0.110 and 0.275, respectively; this finding indicated that the strand that encoded genes contained more T and G than A and C, and this skew was evidence of codon usage bias. Among the 13 protein-coding genes (12,063 bp) of the Yimeng scorpion, there is only three genes (*ND1*, *COX2*, *COX3*) begin with the common start codon “ATT”, 4 (*ND4*, *ND4L*, *CytB*, *ND2*) with “ATG”, 3 (*ND6*, *COX1*, *ATP8*) with “TTG”, 1 (*ND5*) with “ATC” and 2 (*ND3*, *ATP6*) with “ATA”. For termination codons, the seven protein-coding genes (*ND4L*, *ND1*, *ND3*, *CytB*, *COX1*, *ATP8*, *ATP6*) terminated with “TAA”, then the remaining genes ended with incomplete stop codon T–– or AT. Except for *ND6* and eight tRNA genes (*tRNA-Gln*, *Ala*, *Asn*, *Cys*, *Tyr*, *Ser*, *Glu*, *Pro*), all the mitochondrial genome genes were encoded on the H strand. The *12S rRNA* (784 bp) gene and *16S rRNA* (1367 bp) gene were located between the *tRNA-Gln* and *tRNA-Leu* genes, and separated by the *tRNA-Val* gene, with the same result as other arthropods. In addition, the small non-coding region, a putative control region, was located between *12S rRNA* and *tRNA-Gln* genes with the length of 781 bp. Due to lack of *tRNA-Asp*, the Yimeng scorpion mitochondrial genome encodes only 21 tRNA genes, which has not been observed in any other non-scorpion mitochondrial genome published to date (Hwa Choi et al. 2007), and the length of 21 tRNA were between 54 and 70 bp. Thus, data would be helpful to further investigations of phylogenetic relationships within Buthidae.

**Figure 1.**
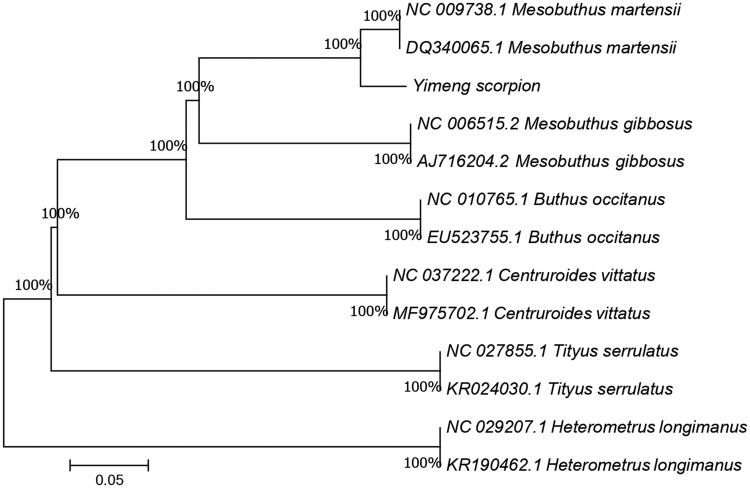
A phylogenetic tree constructed based on the comparison of mitochondrial genome sequences of the Yimeng scorpion (*Mesobuthus martensii*) and other five species of Buthidae family. They are *Mesobuthus martensii* (Chinese scorpion), *Mesobuthus gibbosus* and *Buthus occitanus* (both are Mediterranean scorpion), *Centruroides vittatus* (American scorpion), *Tityus serrulatus* (Brazilian scorpion), *Centruroides limpidus* (South American scorpion). *Heterometrus longimanus* are using as outgroup. Genbank accession numbers for all sequences are listed in the figure. The numbers at the nodes are bootstrap percent probability values based on 1000 replications.
